# Laparoscopic liver hanging maneuver through the retrohepatic tunnel on the right side of the inferior vena cava combined with a simple vascular occlusion technique for laparoscopic right hemihepatectomy

**DOI:** 10.1007/s00464-017-6007-x

**Published:** 2017-12-21

**Authors:** Hongpeng Chu, Guojun Cao, Yong Tang, Xiaolong Du, Xiaobo Min, Chidan Wan

**Affiliations:** Department of Hepatobiliary Surgery, Union Hospital, Tongji Medical College, Huazhong University of Science and Technology, Wuhan, 430022 China

**Keywords:** Laparoscopy, Hepatectomy, Hemihepatectomy, Vascular occlusion, Liver hanging maneuver, Goldfinger Dissector

## Abstract

**Background:**

Laparoscopic hepatectomy has been performed in many hospitals, with the development of the laparoscopic operation technique. However, performing complex laparoscopic hepatectomy, such as right hemihepatectomy, is still a challenge. The aim of this study was to describe the application of a simple vascular occlusion technique and new liver hanging maneuver (LHM) in complex laparoscopic hepatectomy, which are both advocated by Chen Xiaoping for open hepatectomy.

**Methods:**

The clinical data of 29 consecutive patients who underwent laparoscopic right hemihepatectomy (LRH) from October 2014 to October 2016 were retrospectively analyzed. During operation, the vascular occlusion technique without hilus dissection and LHM through the retrohepatic avascular tunnel on the right side of the inferior vena cava were used.

**Result:**

All 29 operations were successfully performed laparoscopically, while adopting Chen’s methods. The study consisted of 23 patients with hepatocellular carcinoma, four patients with intrahepatic cholangiocarcinoma, and two patients with hepatic metastasis of colonic carcinoma. The tumor size was 12.4 ± 1.9 cm. The operation time of LRH was 190.3 ± 49.9 min. The intraoperative blood loss of LRH was 281.7 ± 117.8 mL; five patients required blood transfusion, and the amount of blood transfusion was 300.0 ± 89.4 mL. No case was converted to open surgery, and no death occurred. All resulted in R0 resections. The median free margin was 20.1 ± 10.8 mm. The time of postoperative oral diet intake was 2.10 ± 0.96 days. The complication rate was 17.2%. The average hospital stay after operation was 10.0 ± 2.9 days.

**Conclusion:**

Complex hepatectomy is a bloodless procedure that can be performed under a laparoscope safely using Chen’s methods of vascular occlusion technique and LHM.

Liver resection is the main treatment option for hepatic carcinoma. Laparoscopic liver resection (LLR) could yield the same therapeutic effects with microincision, less trauma, less pain, and quicker recovery [1–3]. More LLRs were performed with the application of advanced techniques and instruments in LLR since the first LLR was reported by Reich in 1991. Laparoscopic left lateral sectionectomy has been regarded as a standard treatment option [4–6]. However, laparoscopy is not widely accepted for liver resections, especially in complex hepatectomies, such as right hemihepatectomy, because of the difficulty associated with controlling bleeding and exploring the deeper region of the liver. Because of these difficulties, LRH has not still become a standard treatment option.

During open hepatectomy, Chen Xiaoping, a Chinese professor, devised a simple vascular occlusion technique and a new LHM [7, 8] different from the traditional LHM advocated by Belghiti et al. [9, 10] for controlling bleeding and exploring structures, which yielded good effects. This vascular occlusion technique ligated the right hemihepatic pedicle (RHP) en masse rather than performed the ligation of the vessels and bile ducts, respectively. Further, this hanging maneuver, which could allow exploration and bleeding control from the hepatic transection plane, can be safely and easily implemented, since the retrohepatic tunnel on the right side of IVC is avascular. It should be suited to LRH theoretically because of its simplicity and usefulness. By applying these techniques in LRH, we performed these operations successfully.

## Materials and methods

### Patients

Between October 2014 to October 2016, 29 consecutive patients with malignant tumors underwent LRH at the Hepatobiliary Surgery Center, Union Hospital, Tongji Medical College, Huazhong University of Science and Technology, China. Preoperative imaging and laboratory examinations were performed for the assessment of tumor size and location and liver function and operation planning, which included computer tomography (CT) and/or magnetic resonance imaging (MRI); three-dimensional reconstruction; hepatitis B and C virus serology; test for tumor markers, including alpha fetoprotein (AFP), carcinoembryonic antigen (CEA), and carbohydrate antigen19-9 (CA19-9); and other routine biochemical tests. Preoperative decision-making for LRH was based on tumor size and location and future liver volume (FLV)/standard liver volume (SLV) calculated by three-dimensional reconstruction.

The Ethics Review Board of Wuhan Union Hospital approved this study.

### Selection criteria

The selection criteria were as follows: (1) single tumor located at more than two hepatic segments of V, VI, VII, VIII without intrahepatic metastasis; (2) no tumor invasion in the first and second hepatic portals and IVC; (3) at least a Child–Pugh B level of liver function; (4) less than 15% indocyanine green 15 min-retention rate (ICG R15); (5) no history of abdominal surgery; and (6) no serious organ damage.

### LRH procedures

**Fig. 1:**
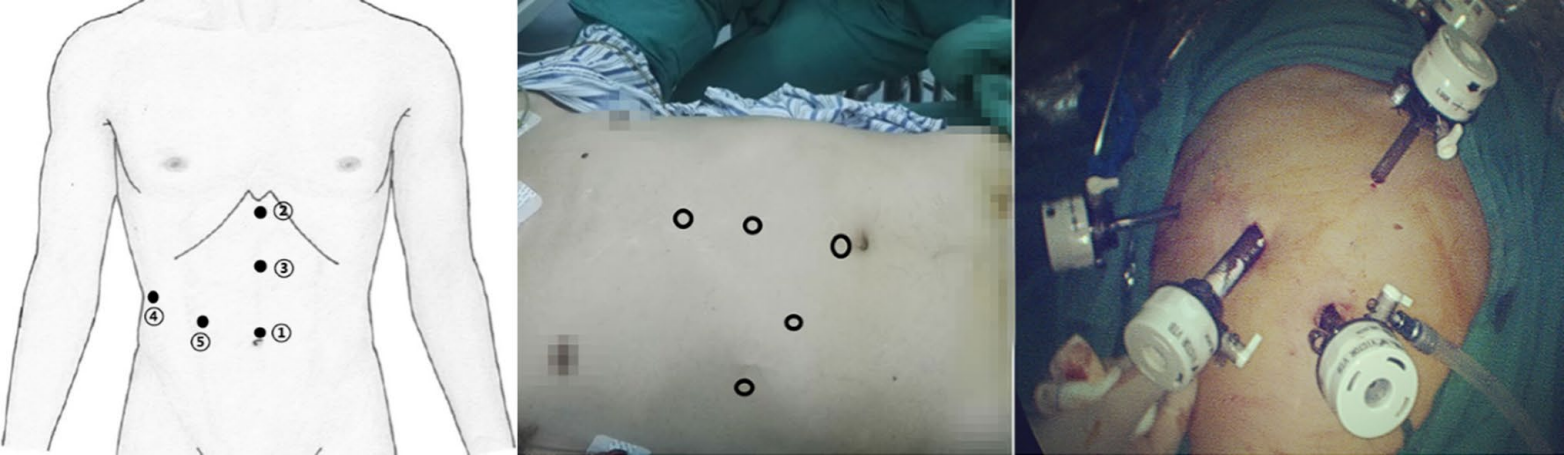
Trocar sites. *①* Above the navel (10 mm); *②* below the xiphoid process (12 mm); *③* between the previously mentioned two locations (5 mm); *④* below the right rib margin and along anterior axillary line (5 mm); *⑤* between first and fourth location (12 mm)

We used five trocars for the operations. Their puncture locations are shown in Fig. 1.

**Fig. 2:**
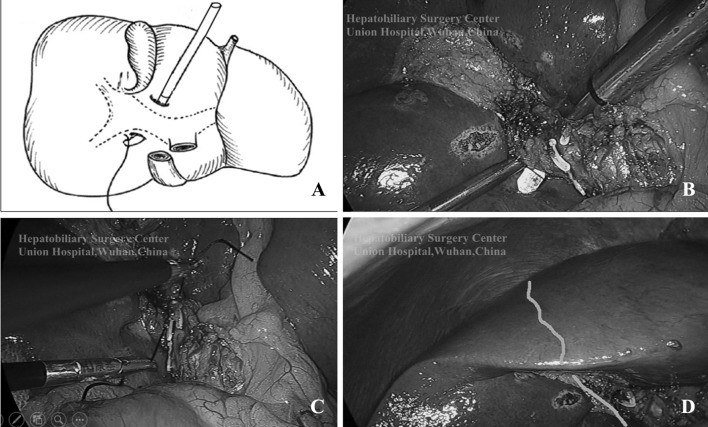
Ligating the RHP. **A, B** Insert a flat and cambered Goldfinger Dissector into the hepatic parenchyma Overriding the Glisson’s sheath of the right hemihepatic pedicle; **C** A No.0 suture is pulled through the tunnel to ligate the right hemihepatic pedicle en masse; **D** The ischemia line is obvious after occlusion of hemihepatic pedicle

**Fig. 3:**
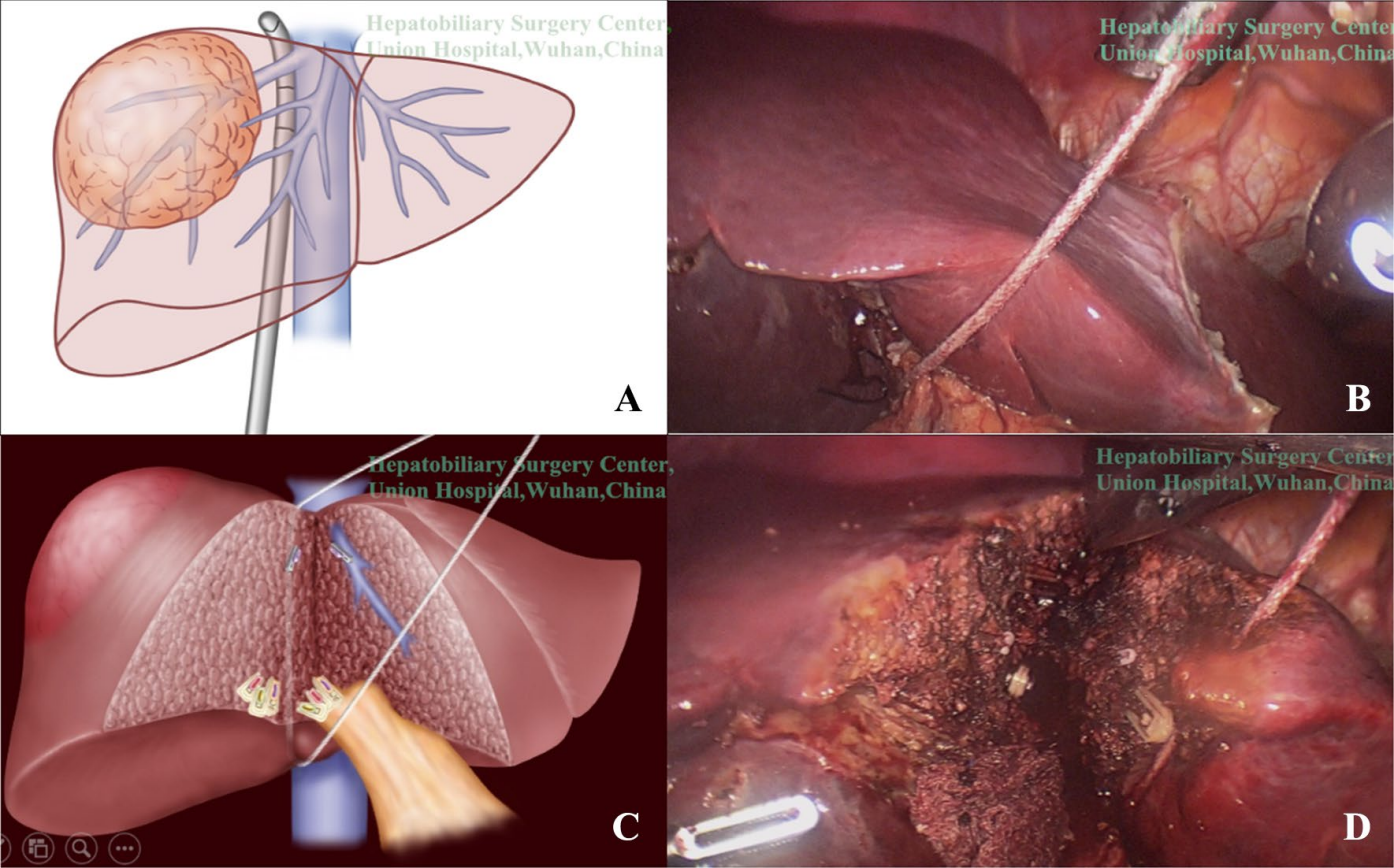
Building the liver hanging maneuver. **A** The Goldfinger Dissector passing through the retrohepatic space along the right side of the suprahepatic IVC and penetrating out from the right side of RHV; **B** A tape was pulled through the retrohepatic tunnel for hanging the liver. **C, D** The liver transection is guided by the hanging tape

Operating procedure was as follows: (1) The teres ligament, falciform ligament, and right coronary ligament were dissected 3–5 cm. Intraoperative ultrasound was performed routinely to ensure that no metastases were found in the future liver remnant and enough resection margin. (2) The gallbladder was routinely excised. (3) A tape was placed around the duodenohepatic ligament and the infrahepatic IVC, respectively, to ligate the first porta hepatis and IVC for bleeding control, if necessary. (4) The RHP was ligated by using Chen’s vascular occlusion technique (Table 1; Fig. 2). (5) The LMH was built (Table 2; Fig. 3). (6) The plane of liver transection was determined by the ischemia line and intraoperative ultrasound and guided by the hanging tape. The Endo Gia stapler was used for dividing the RHP and right hepatic vein during transection of the hepatic parenchyma. (7) The hepatorenal, right coronary, and triangular ligaments were mobilized and divided. (8) The part of the liver to be removed was extracted via an 8-cm suprapubic incision.

**Table 1:** Steps of ligating the right hemihepatic pedicle by Chen’s vascular occlusion technique

Order	Steps
①	Inserting a flat and cambered Goldfinger Dissector into the hepatic parenchyma 2–3 cm without dissecting the connective tissue on the surface of the hilar transverse fissure at the right base of segment IV, about 1.0–1.5 cm right of the margin of the gastroduodenal ligament
②	Overriding the Glisson’s sheath of the right hemihepatic pedicle, then guiding the dissector towards the right posteroinferior parenchyma, finally penetrating the parenchyma at the caudate process of the right inferior margin of the hilar transverse fissure
③	A no.0 suture is pulled through the tunnel by the Goldfinger Dissector. Then ligating the right hemihepatic pedicle en masse rather than performing ligation of the vessels and bile ducts, respectively

**Table 2:** Steps of building the liver hanging maneuver

Order	Steps
①	Dividing the peritoneum on the right side of the IVC just inferior to the liver to expose the right adrenal gland
②	Dissecting the space from below upward between the hepatic parenchyma and the anterior and superior edge of the right adrenal gland, and then along the right side of the IVC. Then the retrohepatic space is dissected at the right of right hepatic vein (RHV)
③	Inserting a cylindrical cambered Goldfinger Dissector passing through the retrohepatic space and arriving at the right side of the suprahepatic IVC
④	A tape was pulled through the retrohepatic tunnel for hanging the liver

### Statistical analysis

All analyses were performed using SPSS 17.0 software. Numerical data were expressed as $\bar {x} \pm {\text{s}}$ .

## Result

The patients consisted of 23 men and 6 women. Their median age was 51.8 ± 10.6 years (range 29–73). The Child–Pugh level was Child–Pugh A in 28 patients and Child–Pugh B in one patient. The ICG R15 result of all the 29 patients was less than 15%. Liver cirrhosis was found in 20 patients (19 due to hepatitis B virus and one due to hepatitis C virus). Another two patients had a positive hepatitis B virus finding without liver cirrhosis. The histological diagnosis was hepatocellular carcinoma in 23 patients, intrahepatic cholangiocarcinoma in four patients, and hepatic metastasis of colonic carcinoma in two patients. The time of the first postoperative flatus was 2.10 ± 0.96 days. The details are shown in Table 3.

**Table 3:** Patient demographics and clinical data (*n* = 29)

Characteristics	Value
Age (years)	51.8 ± 10.6 (29–73)
Sex ratio (M:F)	23:6
Child–Pugh grade	
A	28 (97%)
B	1 (3%)
Cirrhosis	
Yes	20 (69%)
No	9 (31%)
Preoperative laboratory examinations	
HBsAg positive	22 (76%)
Anti-HCV positive	1 (3%)
AFP positive	16 (55%)
CEA positive	3 (10%)
CA19-9 positive	4 (14%)
CA125 positive	7 (24%)
Hemoglobin (g/L)	128.9 ± 21.7
Total bilirubin (μmol/L)	16.1 ± 6.9
ALT (U/L)	54.2 ± 54.3
AST (U/L)	57.9 ± 48.2
Prothrombin time (s)	13.7 ± 1.2
ALB (g/L)	39.3 ± 6.0
ICG-R15 (%)	5.8 ± 2.6
Remnant functional/standard liver volume (%)	47.7 ± 4.3
Histological diagnosis	
HCC	23 (79%)
IHC	4 (14%)
HMCC	2 (7%)

Values are expressed as mean ± SD or number (%)

*HBs Ag* hepatitis B surface antigen, *Anti-HCV* hepatitis c virus antibody, *AFP* alpha fetoprotein, *CEA* carcinoembryonic antigen, *CA19-9* carbohydrate antigen 19-9, *CA125* carbohydrate antigen 125, *ALT* alanine aminotransferase, *AST* aspartate aminotransferase, *ICG-R15* indocyanine green retention rate at 15 min, *HCC* hepatocellular carcinoma, *IHC* intrahepatic cholangiocarcinoma, *HMCC* hepatic metastasis of colonic carcinoma

All the 29 operations were successfully performed laparoscopically. The tumor size was 12.4 ± 1.9 cm, and the resection margin was 20.1 ± 10.8 mm. The operation time for LRH was 190.3 ± 49.9 min. The duration of RHP ligation was 10.0 ± 3.0 min, and that of laparoscopic LHM was 30.8 ± 10.3 min. The intraoperative blood loss volume during LRH was 281.7 ± 117.8 mL. The success rate of retrohepatic tunnel dissection and hemihepatic pedicle occlusion was 100%. Five patients required blood transfusion, and the amount of blood transfusion was 300.0 ± 89.4 mL. The details are shown in Table 4.

**Table 4:** Intraoperative data (*n* = 29)

Characteristics	Value
Duration of operation (min)	
Total	190.3 ± 49.9
Ligating RHP	10.0 ± 3.0
Laparoscopic LHM	30.8 ± 10.3
Blood loss (mL)	281.7 ± 117.8
Patients required transfusion	5 (17%)
Transfusion (mL)	300.0 ± 89.4
Conversion	0
Tumor size (cm)	12.4 ± 1.9
Resection margin (mm)	20.1 ± 10.8

Values are expressed as mean ± SD or number (%)

The complication rate was 17.2%. Pleural effusion combined with ascites occurred in one patient who was treated with thoracentesis for drainage (grade IIIa). Another pleural effusion in one patient was managed without surgical intervention (grade I). One patient had pneumonia, and two had refractory ascites. All of them were treated with pharmacological treatment. No postoperative bile leak occurred (grade II). The average hospital stay after operation was 10.0 ± 2.9 days. The perioperative mortality rate was 0. The details are shown in Table 5.

**Table 5:** Postoperative data (*n* = 29)

Characteristics	Value
complication	5 (17.2%)
Clavien–Dindo classification	
Grade I	
Pleural effusion	1
Grade II	
Pneumonia	1
Ascites	2
Grade IIIa	
Pleural effusion	1
Reoperation	0
Postoperative time to first flatus (days)	2.10 ± 0.96
Hospital stay (days)	10.0 ± 2.9
Mortality	0

Values are expressed as mean ± SD or number (%). Complications graded according to Clavien–Dindo classification

## Discussion

Laparoscopy has become widely used in surgical operations owing to the microincision, less trauma, less pain, and quick recovery. Reports regarding LLR increased, such as LLRs for benign and malignant tumors, hepatolithiasis, hepatic cyst, and even associating liver partitioning and portal vein occlusion for staged hepatectomy (ALPPS) and living donor liver transplantation [6, 11–16], with the invention of different instruments, application of new techniques, modification of conventional methods, etc. However, complex LLRs, such as LRH, still remain to be explored because of the difficulty associated with bleeding control and exposure during laparoscopy.

Vascular occlusion is crucial for hepatectomy. Pringle was the first to advocate ligating the inflow vessels of the liver during transection of the parenchyma for bleeding reduction [17, 18]. While ischemic damage is a major problem [19, 20], ligation and division of the corresponding vessels and ducts via dissection of the hepatic hilus for blood loss reduction during hepatic parenchyma transection when performing right hepatectomy were first described by Lortat-Jacobs and Robert in 1952, which were known as anatomical hepatectomy or classical hepatectomy later [21]. However, the risk of hemorrhage during dissection and bile leaks occurring in the postoperative period makes this approach not suitable for laparoscopy. Chen and colleagues devised a simple vascular occlusion technique without tedious and time-consuming hilus dissection during open left and right hepatectomies [7]. By applying this technique for LLRs using a Goldfinger Dissector instead of a clamp, we obtained a satisfying result. We did not need to dissect the Glisson’s sheath of the pedicle to ligate the artery, vein, and bile duct. We can control them en masse while avoiding bleeding and injuring the bile ducts during hilus dissection during LRH. It was convenient to perform such from our practice. The duration of the RHP ligation was only 10.0 ± 3.0 min during our operations. The feature of amplifying the view offered by endoscopes can help the operator ligate the hemihepatic pedicle.

In 2001, Belghiti et al. reported a kind of LHM for an easier parenchymal transection at deeper sites, better bleeding control, and shorter duration of transection using a tape to pass through the retrohepatic tunnel between the anterior surface of the IVC and the liver [9, 10]. Since then, LHM has been applied in various anatomical hepatectomy procedures worldwide, even including laparoscopic hepatectomy [10, 22–24]. Several other groups demonstrated a success rate of 94% of dissecting the retrohepatic tunnel blindly [25, 26]. The rate of bleeding during retrohepatic dissection due to an injuring short hepatic vein was 4–6% as reported [27, 28]. Chen et al. modified the hanging maneuver in open right hepatectomy [8]. From our practice, we found that Chen’s approach is very suitable for LLRs. First, the retrohepatic tunnel dissected at the right side of the IVC is avascular, which is the crucial difference from Belghiti’s approach. Studying the anatomy of the liver, we can find that the retrohepatic right lateral IVC region consists of connective tissues, and the entire caudate lobe is located at the left side of this tunnel. In other words, this area is a part of the bare area of liver at the right side of IVC, which is avascular. The risk of bleeding during establishment of the tunnel is very low. However, the tunnel in Belghiti’s approach passes through the anterior surface of the IVC. The short hepatic veins of the third porta of the liver, especially those drained from the caudate lobe, existed in this area [29, 30]. The risk of injuring the IVC and its branches increased. The tapes pass through the right side of the right hepatic vein (RHV) instead of between the RHV and middle hepatic vein (MHV) in Belghiti’s approach, so that there is less possibility to injure the RHV and MHV during dissection. Second, the tunnel was established via blunt dissection using the Goldfinger Dissector, which guaranteed lesser bleeding risks. Third, the important point is that we can have a better view at the retrohepatic space using the laparoscope during hanging, while tunnel dissection is performed blindly owing to a poor retrohepatic view in open surgery. During laparoscopic surgery, we can approximately visualize the tunnel (Fig. 3). The duration of this process was 30.8 ± 10.3 min. Further, there was no severe bleeding during our tunnel development. However, our suggestion for those willing to use this method is that they should avoid positioning the tunnel incorrectly. The right adrenal veins may be injured if the tunnel is established at too later position, which may lead to severe bleeding.

Based on these results, all the 29 operations were performed successfully using laparoscope without conversion. Only five patients needed transfusion (300.0 ± 89.4 mL). Further, the duration of the two processes was short. No bile leakage occurred, since the risk of bile duct injury was avoided with these approaches. Two patients suffered refractory ascites. The main reason may be that the FLV/SLV was too low (40.5 and 41%).

In conclusion, LRH can also be performed feasibly, easily, and safely via the application of this new LHM and the simple vascular occlusion technique, owing to the satisfying outcomes of short operation time, less blood loss, and microincision, consequently providing good recovery. We believe that LRH may also be regarded as a standard treatment option with the application of Chen’s methods.
